# Walking the walk? Experiments on the effect of pledging to vote on youth turnout

**DOI:** 10.1371/journal.pone.0197066

**Published:** 2018-05-29

**Authors:** Mia Costa, Brian F. Schaffner, Alicia Prevost

**Affiliations:** 1 Department of Political Science, University of Massachusetts Amherst, Amherst, MA, United States of America; 2 Environmental Defense Fund, Washington, DC, United States of America; Brigham Young University, UNITED STATES

## Abstract

Psychological theories of political behavior suggest that commitments to perform a certain action can significantly increase the likelihood of such action, but this has rarely been tested in an experimental context. Does pledging to vote increase turnout? In cooperation with the Environmental Defense Fund during the 2016 election, we conduct the first randomized controlled trials testing whether young people who pledge to vote are more likely to turn out than those who are contacted using standard Get-Out-the-Vote materials. Overall, pledging to vote increased voter turnout by 3.7 points among all subjects and 5.6 points for people who had never voted before. These findings lend support for theories of commitment and have practical implications for mobilization efforts aimed at expanding the electorate.

## Introduction

Voting is one of the most important civic opportunities offered to citizens, yet the persistent low participation rates of young people have long puzzled scholars, policymakers, and activists concerned with cultivating an active electorate. Ever since 18-year-olds were first given the right to vote in 1972, the youth vote has consistently lagged behind that of older generations. In 1972, 51% of 18-to 24-year-olds voted compared to 70% of people who were 25-years-old and older. The age gap in voter turnout was not much better in 2016, with turnout levels at 50% for young people and 65% for those above 25 years old [1]. Young citizens may vote at lower rates than other Americans for a variety of reasons. Young people have fewer resources that are traditionally thought to increase one’s participation, such as education and income [2]. The informational costs of finding out how and where to register to vote and cast one’s ballot can deter participation for some newly eligible voters [3, 4]. Indeed, there is some evidence that suggests allowing young citizens to register to vote before they are actually eligible—thus diminishing their chances of missing registration deadlines—can increase youth turnout [5]. Moreover, the lack of geographic stability of many young people poses additional costs for voter registration and turnout [6].

Political scientists have long argued that mobilization is a key factor in getting people to turnout to vote. Recent studies using field experiments have shown how effective grassroots tactics can be in mobilizing people to vote [7, 8], but turnout among young citizens has not been widely studied. Few mobilization campaigns focus specifically on young people, in part because of the low returns anticipated with targeting America’s least active portion of the electorate. One technique that has become more common in campaigns’ repertoire of mobilization techniques, especially to boost youth voter turnout [9], is the use of vote pledges. The intuition behind this mobilization tactic is simple: if a person commits to a future behavior, they will be more likely to follow through on that behavior than someone who was not asked to commit.

Does committing to participate increase political engagement? Specifically, we are interested in whether pledging to vote increases the likelihood that an individual will turn out to vote. Research on cognitive psychology suggests that commitments to perform a certain action can significantly increase the likelihood of such action, but this has rarely been empirically tested in a political context. While pledging is a relatively simple mobilization tactic, it invokes basic psychological processes above and beyond the traditional demographic parameters in models of voter turnout, such as education, race, and age [10]. Theories of commitment, cognitive dissonance, and self-perception suggest that pledging to vote may engage a whole host of psychological mechanisms that explain how individuals can be persuaded by their own actions.

In cooperation with the Environmental Defense Fund (EDF), first during the 2016 Pennsylvania primary election and second during the general election campaign in Colorado, we conduct the first randomized controlled trials testing whether individuals who pledge to vote are more likely to turn out than individuals who are contacted using standard Get-Out-the-Vote (GOTV) materials, such as reminders and information about the election. Overall, we find that pledging to vote had a significant impact on turnout among individuals who had not previously voted. The Pledge to Vote campaign was particularly effective for bringing new voters into the electorate in large numbers, rather than just ensuring turnout among regular voters. These findings suggest that new voters are more susceptible to commitments to participate in politics and have practical implications for increasing youth turnout.

## The psychological bases of voting behavior

Several different psychological theories of behavior suggest that “talking the talk” can lead individuals to “walk the walk.” Applying these theories to voter turnout critically hinges on conceiving of voting as a dynamic set of behaviors that extend over time, rather than one static act [11]. Events that occur before a person decides to vote, including processes of cognition, can influence whether or not they follow through and actually cast a vote. Our particular prebehaviorial theory of voting departs from the view that voting serves an instrumental, electoral function for quasi-rational individuals (i.e. that their individual vote will affect the election outcome), and instead treats voting as a cognitive process and an act of self-expression.

First, theories of self-prediction suggest that if people expressly predict that they will behave a certain way, then they are more likely to follow through on that behavior. This phenomenon is described in different literatures as the “self-erasing nature of errors of prediction” [12], the “self-prophecy effect” [13], and the “mere measurement effect” [14]. Applied to a GOTV context, the expectation is clear: a person is more likely to vote if they had previously *predicted* that they would do so. The most recent empirical evidence on the effectiveness of voting self-predictions, however, is more mixed. In an analysis of an experiment conducted during the 2000 presidential primary, Smith, Gerber, and Orlich [15] find that neither being asked to predict whether one would vote or to provide an “important reason” for voting increased one’s likelihood of turning out to vote. Nickerson and Rogers [16] additionally find that during the 2008 Pennsylvania primary, self-predictions had a statistically significant but weak effect on turnout. Combining the self-prediction ask with a voting implementation plan was more effective; that is, individuals who were asked to predict whether they would vote and also to answer a series of questions regarding their plan on election day (i.e. what time they will vote, where they will be coming from, etc.) were significantly more likely to turnout to vote than those who were only asked to predict whether or not they would vote.

One reason why voting plans are more effective than self-predictions alone is because verbalizing implementation intentions mimics a sort of *commitment*. There is a long-standing body of literature that suggests individuals will stick to a previous commitment—especially if they receive a reminder of that commitment—in order to avoid feelings of inconsistency and cognitive dissonance between their expressed intentions and behavior [17–20]. Cognitive dissonance is foundationally described as a mental state that occurs in the context of a “belief dilemma”: the uncomfortable psychological conflict between two or more beliefs [17]. Applied to an action-based model, cognitive dissonance can influence people to act in a certain way that accords to their attitudes [21, 22]. When actions do not match cognition, dissonance occurs.

Self-perception theory developed in contradiction to theories of cognitive dissonance, yet both are related in explaining one’s propensity to follow through on a prior commitment. Self-perception theory suggests that attitudes are induced from behavior, rather than the other way around [23, 24]. Individuals develop and establish their attitudes by observing their actions and inferring what attitudes must have led to those actions. Therefore, initial commitments can change an individual’s self-image, thereby promoting later behaviors that align with that self-image. Appealing to this behavioral pattern is known as the foot-in-the-door technique [20, 25]. Once an individual complies with an initial request (i.e. signing a pledge to vote card), they will be more likely to comply with a subsequent, larger request (i.e. voting). This is in contrast to the door-in-the-face technique [26], where someone first rejects an initial request that is too extreme, resulting in feelings of obligation that cause them to agree to a second, smaller request. Notably, McCabe and Michelson [27] test how effective this technique is in turning people out to vote and find that simply asking people if they could be counted on to vote is much more effective. Additional evidence suggests that follow-ups and reminders of one’s prior behavior can significantly increase the likelihood of subsequent action [28, 29].

Indeed, explicitly committing to a particular way of acting adds a public element to one’s perceived sense of self. In this sense, self-perceptions not only internally drive people to act a certain way, but can also increase the perceived *social* costs of failing to fulfill that self-perception [30]. Commitments activate a basic psychological desire in people to behave in manner that is consistent with both their own self-perceptions and the social norms attributed to those perceptions [23, 24, 30]. Pledging to vote, for example, may invoke self-perceptions of oneself as a “voter.” Social identity theories suggest that a person’s self-esteem increases when they behave in ways that are consistent with the behavior of in-groups with which they identify [31], and this pattern becomes stronger as the norm of voting becomes more salient in one’s social network [32]. Therefore, being reminded of one’s pledge to vote may contribute to a person’s identity as a voter and the accompanied social norm that voting is important.

One example of the power of pledges to invoke dissonance is the myriad of behaviors individuals have been found to engage in to maintain consistency. Not only do people express attitudes that accord with their behaviors [23], but if a behavior is inconsistent with prior attitudes and commitments, people may alter their perceptions about the prior behavior. For example, in a longitudinal study of middle- and high-schoolers, 82% of those who pledged abstinence but then engaged in sexual activity denied ever having taken a pledge of abstinence in the first place [33].

We therefore expect people who pledge to vote to be more likely to turn out because they observe and recall their act of pledging, and thus infer that voting is an important element of their self-image and identity. It is this self-induced attitude about the importance of voting that would then lead to cognitive dissonance if the commitment to vote was not adhered to. Therefore, we posit that both self-perception and cognitive strategies to avoid dissonance would lead people who pledge to vote to turn out to vote on election day.

However, we expect that there will be differential effects among people who vote regularly, people who regularly do *not* vote, and people who are “on the cusp of voting” [34, 12] such as young adults. Specifically, we expect that those in that latter category should be more susceptible to the theories of self-perception and cognitive dissonance outlined above. Behaviors are most likely to translate into attitudes when attitudes about the behavior have yet to be fully crystallized [23]. Therefore, if pledging does invoke self-perceptions about voting, the effects of the pledge campaign should be the most pronounced for people who have not yet created an identity around the act of voting. People who have not experienced the cognitive process of identifying as a voter, non-voter, or occasional voter, are the ones that have the most to “gain” (i.e. are the most likely to see a change in their behavior).

Notably, young adults become eligible to vote at the same time that they are particularly “vulnerable” to attitudinal change. The impressionable years hypothesis asserts that young people are more open to persuasion when it comes to attitudes and behaviors in part because they are in the midst of forming a self-identity and establishing the behaviors associated with that identity [35, 36]. For these reasons, young adults are a group who may be especially influenced by pledging to vote, and the impact of such effects may persist over the long term. After all, the habitual voting literature argues that once an individual votes it is likely that she will turn out to vote in subsequent elections [37–41]. Thus, the earlier that eligible voters get to the polls, the more likely that we will see higher levels of general participation. This suggests that young people are not only an especially persuadable group of voters, but they are also a group for which a single act of mobilization can spur a lifetime of turnout.

Despite the rich theoretical body of literature that suggests pledging can increase the likelihood of a behavior, surprisingly little has been done to empirically test this theory, especially in political contexts. Much of the research on the power of pledges to impact behavior has studied non-political outcomes. For example, individuals who pledge to recycle have been found to be significantly more likely to do so than individuals who did not pledge to recycle [42]. Similar effects have been found for wearing seat belts [43] and safety glasses [44]. These studies, however, are relatively under-powered and future research is needed to better understand the effect of pledging on these behaviors.

One study analyzed data from a 1996 Rock the Vote campaign that used pledge reminders to increase voter turnout [9]. However, there are three limitations to the ability to draw strong causal inferences from this study. First, the authors did not investigate whether pledges increased turnout over other GOTV tactics, such as simply encouraging people to vote and providing information about the election, but rather whether a personalized pledge was more effective than a generic pledge. Second, the study was not a randomized controlled trial but rather an analysis of observational data. Third, the authors rely on self-reported measures of turnout for their outcome variable instead of using validated vote records. Our study seeks to extend and improve upon this work by implementing two randomized controlled trials to examine the effect of pledging on turnout in comparison to traditional GOTV tactics. By administering pledges randomly, we can draw out clear causal effects of the campaign. Additionally, by using validated voter turnout, we avoid the limitations of self-reported turnout—specifically, the tendency of individuals to lie about voting.

## Experimental design

Human subject research approval came from the University of Massachusetts Amherst Human Research Protection Office, Protocol ID 2017-3629. The Pledge to Vote campaigns in both Pennsylvania and Colorado were designed to determine whether having individuals pledge to be a voter in an upcoming election would make them more likely to vote compared to traditional mobilization techniques, such as reminders and encouragement to turn out. Note that our control is not a “true control” group of people who were not contacted at all. Rather, we are comparing the effectiveness of the pledge compared to a petition among people who were contacted. This is akin to an A/B test and follows the approach of many randomized controlled trials where the point of interest is the effectiveness of various interventions in comparison to one another [45, 46]. Working with Defend Our Future (DOF), a campaign of Environmental Defense Fund (EDF), we designed a field experiment to measure the effectiveness of a pledge to vote program that Defend Our Future was already planning to run in Pennsylvania and Colorado. These states were chosen by DOF because a) they were swing states particularly important to the election, and b) implementation was aided by DOF’s grassroots operations already on the ground in those areas. Given the nature of the organization, the campaign was focused around a message involving climate change.

### Study 1: Pennsylvania

During March and April of 2016, DOF staff recruited individuals on five college campuses in the Philadelphia area (Drexel University, University of Pennsylvania, St. Joseph’s University, Temple University, and Villanova University). These particular campuses were ideal for our purposes because they varied in terms of the student population size and demographics. For example, Temple University is a relatively racially diverse public university with over 36,000 undergraduate students, whereas St. Joe’s is a mostly white, private Jesuit university of less than 9,000 undergraduate students.

The Pennsylvania mobilization effort was strictly nonpartisan—DOF did not support any candidate in the elections. All of the funding for the effort was tax deductible (501c3), which, by regulation, could not be used for partisan political activity. The nonpartisan identity of this campaign enabled us to examine the effect of pledging absent any partisan cues. It also positioned DOF as an alternative to hyper-partisan independent expenditure groups who were already on the ground in Pennsylvania.

DOF had a team of more than a dozen people in Pennsylvania that included a field director, field organizers, volunteers, and interns. The pledge to vote program was an extension of the grassroots organizing work that the DOF organizing team had been conducting on Pennsylvania campuses since the beginning of the 2015-16 school year. For the experiment, DOF organizers recruited individuals to participate through a variety of campus-based communications including: canvassing in high-traffic areas on campuses, presenting in front of classes, reaching out to student groups and clubs, and hosting on-campus social events.

The recruitments happened in 93 shifts of approximately 4 hours each across the course of more than one month. We randomly assigned each shift so that all individuals contacted during a particular shift would either be asked to sign a petition form indicating they would like to be reminded about the election or to sign a pledge to vote. On both cards, individuals filled out their name, address, and contact information. Both the petition (control) cards and pledge (treatment) cards are shown in S1 Fig. In total, 4,110 individuals received the “pledge to vote” contact and 1,655 received the generic turnout contact, where they simply received information about the election. This imbalance was by design; DOF believed that the pledge to vote appeal would be more effective and thus we worked with them to minimize the size of the control group. Thus, we assigned shifts to the control condition with a probability of 0.3 and to the pledge treatment with a probability of 0.7.

All individuals who were contacted were sent a reminder to vote in the mail during the two weeks before the election. Individuals who pledged to vote were sent their pledge while those who signed the petition simply received a generic message encouraging them to vote: “Remember to vote in Pennsylvania’s primary on April 26, 2016.” The pledge card reminder, in contrast, specifically reminded subjects of their pledge: “Remember your pledge to vote in Pennsylvania’s primary on April 26, 2016.”

**Fig 1:**
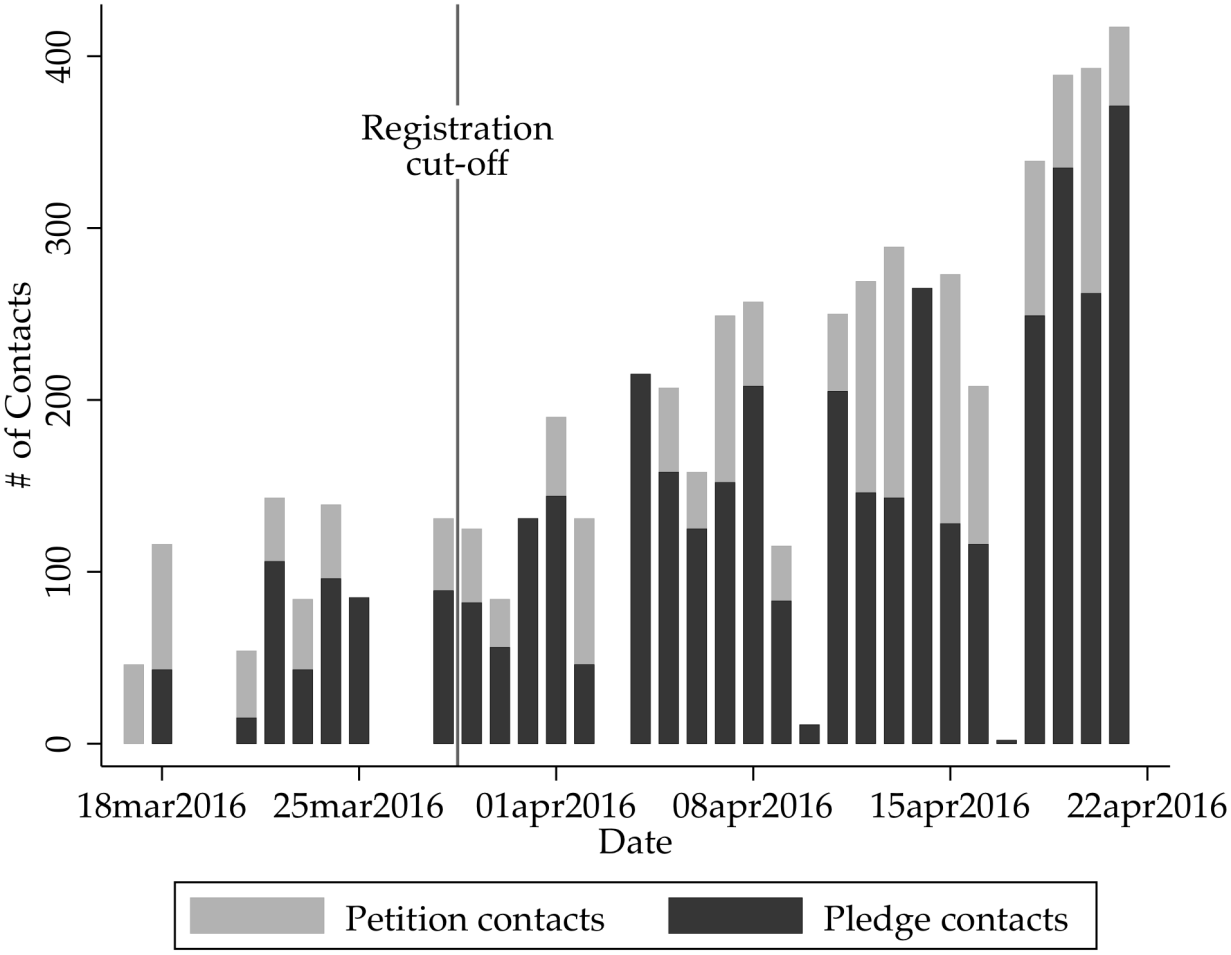
Number of contacts in Pennsylvania.

Fig 1 shows the number of individuals recruited for each day of the campaign in Pennsylvania. One important feature of this program was that for circumstances beyond our control, most of our recruitments happened after the March 28th deadline by which individuals had to register in order to participate in the primary election on April 26th. This clearly did not preclude the organizers from contacting large numbers of individuals, but it did mean that if an individual was not already registered to vote they would not be able to participate in the April 26th primary election.

Field organizers entered contact information for individuals into the Voter Activation Network and we then matched this list to the Catalist database. Catalist is a voter file firm that contains individual-level data on over 240 million voting-age individuals. In Pennsylvania, we were able to successfully match 55% of the records to an individual with an address in Pennsylvania in the Catalist database. Since it is highly likely that someone who was unable to be matched to the voter file is registered to vote, any individual who was unmatched was considered to be a non-voter. This follows the approach recommended by Ansolabehere and Hersh [47] (see also [48]). In addition, our analysis focuses on individuals who could have actually voted as a result of the treatment. This includes all individuals who were contacted before the March 28th registration deadline and anyone contacted after the deadline who had registered to vote before the deadline (thereby making them eligible to participate in the primary).

The nature of the campaign in Pennsylvania is important to consider when examining the effectiveness of vote pledges. In general, turnout in primary elections is significantly lower than in general elections. In 2016, about 33.7% of eligible voters participated in the Pennsylvania primary. In addition, while the Democratic nomination for president was still being contested at the time of the Pennsylvania primary, all challengers to Donald Trump had already withdrawn from the Republican contest. Both of these contextual factors make the Pennsylvania primary election a particularly difficult case for increasing turnout.

### Study 2: Colorado

The second experiment took place during the general election campaign in the state of Colorado. DOF had organizers on the Auraria Campus in Denver where Auraria Community College, Metropolitan State University of Denver, and the University of Colorado Denver all share a campus. They also canvassed on Colorado State University’s campus in Fort Collins and at the University of Colorado campus in Boulder. As was the case in Pennsylvania, organizers recruited individuals by canvassing in high-traffic areas, presenting in front of classes, reaching out to student groups and clubs, and hosting on-campus social events. Shifts in Colorado were randomized in the same manner as those in Pennsylvania—91 shifts (71%) were randomly assigned to the pledge condition while 37 were assigned to the control condition. As with the Pennsylvania experiment, the imbalance in the assignment of shifts to the control and treatment groups was by design.

In total, the organizers recruited 6,176 individuals from September 26 through November 2, 2016: 4,340 pledge to vote cards (70.3%) and 1,836 petition signatures (29.7%). The pledge and petition materials for this study were similar to the ones used in Pennsylvania (See the Supporting Information for images of the treatment and control cards).

There are several advantages to conducting a second experiment, especially in Colorado. First, turnout should not be unequal in a general election swing state along partisan lines, like it may have been in Pennsylvania given the uncontested Republican presidential primary. Second, understanding the effects of pledging to vote in both a primary and general election adds important information about whether this strategy can be applied to different types of elections. In particular, is the pledge to vote tactic more effective in primaries than in more salient general election campaigns?

Third, unlike Pennsylvania, Colorado allows citizens to register to vote up to and on the day of the election. Thus, mobilization efforts that happened closer to the date of the election could still be fully effective, unlike in Pennsylvania where only registered citizens could be mobilized after the registration cut-off date. We therefore include everyone who was contacted by DOF in the analysis.

### Who was recruited?

Before analyzing how pledges influenced turnout, we first elaborate on the demographic profile of individuals who were reached by the organizers. Table 1 shows the composition of these individuals in terms of the percentage successfully matched to the voter file and, among that group, their age, gender, race, partisanship, and vote likelihood. The pledge group and the control group are remarkably balanced. Of particular importance is the average score of recruits on the vote propensity models. These models are created by voter file firms to predict how likely they think an individual is to vote on a score of 0 to 100. In Pennsylvania, the average score for members of the control group was 59.8 while those in the treatment group received an average score of 59.2. The average scores were similarly close in Colorado, with an average of 68.3 for the control group and 69.7 for those recruited to sign a pledge to vote.

**Table 1:** Demographic profile of contacts.

Type	% Matched	Median Age	% Female	% White	Avg. Partisanship	Avg. Vote Likelihood
**Study 1: PA**						
Pledge Group	54%	20	62%	75%	70.6	59.2
N = 4,110						
Control Group	56%	21	61%	74%	72.5	59.8
N = 1,655						
**Study 2: CO**						
Pledge Group	74%	20	61%	84%	64.6	69.7
N = 4,294						
Control Group	77%	21	57%	84%	61.8	68.3
N = 1,783						

Note: Table presents the demographic comparison of contacts in the pledge (treatment) and petition (control) groups for both studies. Data on these measures is available only for individuals successfully matched to Catalist.

Both the pledge program and petition program appeared to be particularly effective in reaching DOF’s target audience of young individuals. The average age among those recruited in Pennsylvania was 24, and the median age was 21. In other words, more than half of those recruited were 21 or younger. For many, this was the first election for which they had been eligible to vote. Likewise, the median age of individuals recruited in Colorado was 20 for the pledge group and 21 for the control group. While the canvassers were located on college campuses to mostly recruit young adults, some people who were contacted were not students. About 12% of the subjects in Pennsylvania for whom we have age information from the voter file were 30 years of age or older. In Colorado, about 8% were 30 or older. In S2 Table, we show that the treatment effects are robust to limiting our analysis to young adults.

### Accounting for shift randomization

As noted above, the nature of the mobilization campaign run by DOF precluded us from conducting individual-level assignment to the treatment and control conditions. Instead, randomization was conducted at the shift level. Accordingly, we need to be attentive to that fact as we analyze the results. Fortunately, DOF’s organizers kept track of the shifts during which each individual was contacted, as well as the canvasser who contacted each individual. In analyzing the results in the following sections, we use cluster robust standard errors to account for clustering by shift. Additionally, in the Supporting Information, we add an analysis in which we control for the canvasser who recruited each individual. We do this to ensure that our results are not a by-product of the particular canvasser who was assigned to each shift. Our findings are robust to this alternative specification.

Finally, an additional concern might be that some individuals would be more likely to sign a petition than a pledge (or vice versa) and that this propensity may be related to their likelihood of voting. While we cannot absolutely rule out this possibility, there are several reasons to think that this is not likely to be a concern. First, during the recruitment, individuals who were approached were only aware of the type of form they were signing well after they began the conversation with the organizer. Thus, by design, there would be no reason to expect that people contacted in one condition would be less likely to at least begin a conversation with an organizer than they would in the other condition. Second, the rate of successful recruitments made during pledge versus petition shifts were almost perfectly in line with what we would expect given how many shifts were assigned to each condition. For example, in Pennsylvania, 69.9% of shifts were assigned to the pledge condition and 71.3% of our subjects signed a pledge to vote. In Colorado, 71.1% of shifts were assigned to the pledge condition and 70.6% of individuals recruited were in the pledge condition. Thus, it does not appear to be the case that one type of shift resulted in higher recruitment rates than the other. These patterns, in conjunction with the balance comparison presented above, help to bolster the expectation that the control and treatment groups were equivalent except with regard to the type of commitment obtained from the subject.

## Results: Study 1

To determine the effectiveness of the pledge campaign, it is necessary to compare individuals who *signed a petition (the control group)* with those who *signed a pledge (the treatment group)*. Table 2 shows several different treatment effects. The first line in the table simply includes every individual who was contacted by DOF. Among the full set of contacts, turnout was quite low and the small difference between the treatment and control groups was in the opposite direction than hypothesized. However, recall that this comparison includes a large number of individuals who could not have been affected by the treatment even if it was persuasive. This is the case because anyone who was contacted after the registration deadline of March 28th was ineligible to vote in the primary unless they had already registered to vote in Pennsylvania.

**Table 2:** Effect of treatment and control on voter turnout—Pennsylvania primary.

Group	Control Group	Pledgers	Difference
All individuals contacted	15.7%	15.1%	-0.6%
	(1.4)	(0.1)	p = 0.721
	N = 1,655	N = 4,110	
All eligible individuals	34.6%	39.1%	+4.5%
	(3.3)	(2.0)	p = 0.199
	N = 751	N = 1,589	
Never voted previously	28.4%	37.2%	+8.8%
	(4.3)	(3.1)	p = 0.057
	N = 457	N = 924	

Note: Cluster robust standard errors (in parentheses) adjust for clustering by recruitment shift.

Accordingly, the second line in the table restricts our comparison to only those individuals who could have actually voted as a result of the treatment. This means that we include all individuals who were contacted before the March 28th registration deadline, as anyone contacted before the deadline could have registered in time to vote in the primary. For those contacted by DOF after the registration deadline, we restrict the subject pool only to those who were already registered to vote. We do this since one could not be affected by the treatment if they were not registered to vote by that date.

The second set of results in Table 2 (“All eligible individuals”) shows the comparison of turnout between those who received the standard mobilization contact and those who were asked to sign a pledge to vote. Among these individuals who could actually have been affected by the contact, people who signed the pledge had a 4.5 percentage point higher chance of voting in the primary than those who signed a petition. Note that the standard errors and p-values in all our analyses are calculated using cluster robust standard errors to account for clustering by the shift during which an individual was recruited.

Of course, the pledge campaign is specifically designed to mobilize individuals who have not previously participated in the electoral process. Thus, the third line of results in the table shows the same turnout comparison, but only for those who had not previously voted. We defined previous non-voters as anyone for whom there was no valid vote record in 2014, 2012, or 2010 (which includes people for whom there were no registration records at all). Among that group, the pledge appears to be more effective. First time voters who signed the pledge turned out at a rate that was 8.8 percentage points higher than first time voters who received the standard mobilization message. The treatment effect among individuals who *had voted* previously was actually—2.4 points (p = 0.533). In a simple OLS model, an interaction term for previous non-voters and the pledge treatment produces an estimated interaction effect of 11.2 points, with p = 0.058.

## Results: Study 2

The Colorado experiment offered an opportunity to examine our treatment effects on a larger scale and in a competitive swing state during the general election. It also removed many of the complications created by the registration deadline in Pennsylvania since Colorado residents can register to vote up to and on the day of the general election. Thus, if an individual was contacted by DOF and provided a Colorado address, then whether they were presently registered or not was not an impediment to them being affected by that recruitment. Accordingly, we include in our analysis everyone who was contacted by DOF, regardless of whether they were matched to the Catalist database. This approach allows us to avoid the concerns about post-treatment bias described by Nyhan et. al. [49].

Table 3 shows the treatment effects for our Colorado study. We show three sets of results. The first line includes in the subject pool every individual contacted by DOF during the campaign, even if they provided an address outside of the state of Colorado. Of the 1,841 individuals who were recruited by DOF to sign a petition, 60.8% were matched to a validated vote record in the state of Colorado. By comparison, of the 4,374 individuals DOF recruited for the pledge to vote condition, 65.0% were matched to a valid record of turnout in Colorado in November 2016. This amounts to a 4.2 percentage point increase in the turnout rate among individuals in the pledge to vote group (p = 0.007).

**Table 3:** Effect of treatment and control on voter turnout—Colorado general election.

Group	Control Group	Pledgers	Difference
All individuals contacted	60.8%	65.0%	+4.2%
	(1.4)	(0.7)	p = 0.007
	N = 1,841	N = 4,374	
All individuals with CO address	62.6%	66.2%	+3.5%
	(1.3)	(0.8)	p = 0.020
	N = 1,783	N = 4,294	
CO address & never voted previously	52.0%	57.0%	+5.0%
	(1.8)	(0.9)	p = 0.015
	N = 1,319	N = 3,212	

Note: Cluster robust standard errors (in parentheses) adjust for clustering by recruitment shift.

A small number of individuals who were contacted in Colorado by DOF provided a home address outside of the state of Colorado. Accordingly, the second set of results in Table 3 limits our analysis to individuals who provided an address in the state of Colorado. Among this group we find a relatively similar treatment effect of 3.5 percentage points (p = 0.020).

Finally, the third set of results in Table 3 focuses on individuals who were likely to have not been previous voters. Our group of previous non-voters includes anyone who was not matched to a record in Catalist as well as those who were matched but for whom we could find no record of turnout in 2016, 2014, 2012, or 2010. We identify unmatched individuals as previous non-voters because the fact that we were unable to find a record for them in Catalist (especially combined with their young age) is likely the result of the fact that they have never been registered to vote. Note that reducing the analysis to previous non-voters only leads to a relatively small reduction in the sample size; this is due to the fact that by focusing the mobilization campaign on young adults, we are already focused on a population that had mostly not voted before. The effect of the pledge treatment was higher among this group—5 percentage points. The treatment effect among individuals who *had voted* previously was just 0.6 percentage points (p = 0.650). In a simple OLS model, an interaction term for previous non-voters and the pledge treatment produces an estimated interaction effect of 4.3 points, with p = 0.070.

### Combined effect from both studies

**Fig 2:**
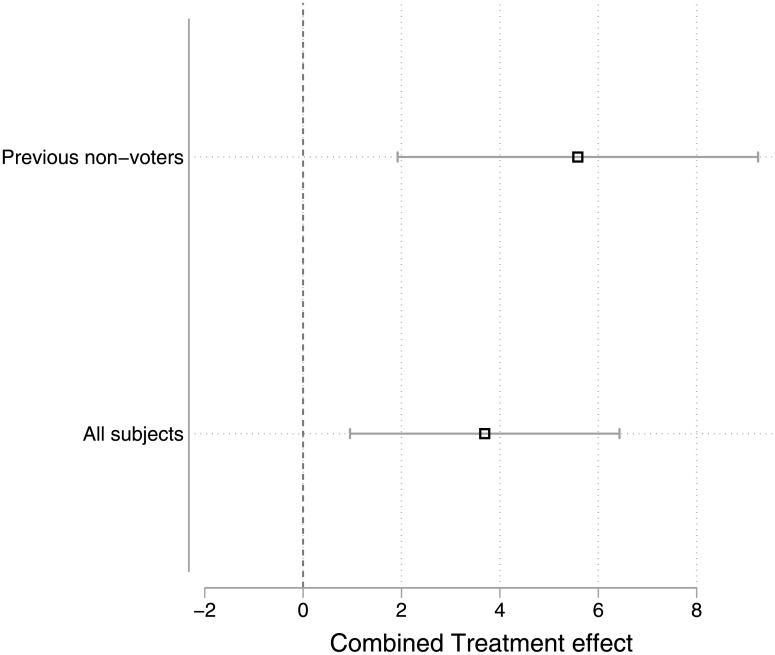
Meta-analysis showing combined treatment effect in both studies.

Given the similarity of the two experiments we carried out in 2016, it is possible to conduct a simple meta-analysis to estimate the combined effect of the pledge to vote treatments across both studies. The meta-analysis approach gives more weight to studies that measure the treatment effect with more precision; thus, in meta-analyzing the Pennsylvania and Colorado experiments, the Colorado study is given about four-times as much weight as the Pennsylvania experiment due to the much larger usable subject size. Fig 2 plots the results from a fixed effects model for all subjects and for previous non-voters. Note that the results are the same using a random effects model.

For all subjects across the two experiments, the effect of signing a pledge to vote rather than a traditional petition was 3.7 points, with a 95% confidence interval ranging from 1 to 6.4 points. For previous non-voters the estimated effect is somewhat larger—5.6 points, with a confidence interval ranging from 1.9 to 9.2 points.

How do these treatment effects compare to other field experiments that test the effects of particular types of messaging on turnout? Green, McGrath, and Aranow [50] summarize the research on message effects in mobilization field experiments. They conclude that “arguments used to encourage voting (civic duty, the closeness of the race, group solidarity) tend to have relatively minor effects, whereas the norms used to frame those arguments (e.g., social pressure, gratitude) have much stronger effects.” As an example of the latter (stronger) effects, Gerber, Green, and Larimer [45] find that individuals exposed to a message that is designed to heighten the social pressure to vote are up to 6.3 points more likely to vote than those who were exposed to a simple “civic duty” message. Other field experiments testing a social pressure message have shown similarly large effects relative to a more basic civic duty message [51]. Thus, our “pledge to vote” intervention appears to have a somewhat more modest sized mobilization effect than has been found for social pressure mailers, though the pledge treatment can be administered without concerns about negative backlash that have been cited for social pressure messaging [52]. In addition, changing from one GOTV message to another (such as a pledge) is effectively costless, so this intervention should not be difficult to implement.

## Discussion

This research contributes to a growing body of work that uses field experiments to understand voter behavior. In this paper, we provide evidence that having individuals pledge to vote is an effective way to increase turnout. This is especially true for individuals who have not previously participated in the political process, such as young adults. From a practical standpoint, this simple mobilization tactic could stimulate increased voter turnout among a relatively inactive segment of the eligible electorate, especially considering the cost to implement a pledge campaign is about the same as any other GOTV campaign. If voting is a habitual behavior [37, 40], getting new voters to the polls could have substantial long-term effects on overall voter turnout.

Our study is a first step at exploring the effect of pledging to vote on turnout. We designed the experiments under a variety of constraints and more experiments should be conducted to test the generalizability of our results to other contexts. For example, future studies should examine the impact of pledges in settings other than college campuses with more diverse target populations. There is also some reason to think that pledging effects may vary by racial or ethnic group since whites, African Americans, and Latinos (for example) all have different voting patterns. Self-perceptions about voting might therefore be enhanced among certain groups, creating lower thresholds for cognitive dissonance for reneging on a pledge to vote. While DOF organizers were able to contact relatively high numbers of non-white individuals in Pennsylvania, after constraining our analysis to those who were registered by the cut-off date, there there were not enough non-whites to uncover statistically significant trends for the effect of pledging to vote for racial or ethnic minorities. Future research should examine this dynamic in more detail.

More research is also needed to examine the impact of vote pledges as utilized through a campaign focused on different issue areas, or a non-issue campaign. The Environmental Defense Fund’s focus on climate change may affect turnout rates because people may self-select into recruitment based on their positions surrounding environmental policies. While this self-selection should be random across our treatment and control groups, thus not biasing the extent to which we can make causal estimates of the effect of the treatment, it would be worthwhile to test whether pledges affect behavior absent their association to a particularized message.

Additionally, research should examine different *types* of pledges. Personalized GOTV appeals have been found to be more successful at mobilizing people to vote than non-personalized appeals [7, 9]. In addition, people are motivated to vote by those in their social networks [32]. Therefore, are pledges that are personalized or appeal the social aspect of voting more effective at increasing turnout than standard pledges? In a similar vein, are there spillover effects of pledging among individuals in the same social network?

Finally, there are a series of unanswered questions regarding other potential outcomes of pledges. Our study is limited to voter turnout, but it is possible that pledges can be used to increase a whole host of other political behaviors. Does pledging make people more likely to attend rallies, put up yard signs, influence others to vote, or write to one’s elected official? Other downstream effects of pledging should also be considered. Does pledging to vote only increase one’s likelihood to vote in the next election, or does a newfound ‘voter identity’ influence people to continue to participate in politics? We look forward to future experiments that take on these questions.

## Supporting information

S1 Fig Study 1: Solicitation materials. (TIFF)

S2 Fig Study 1: Reminder cards. (TIFF)

S3 Fig Study 2: Solicitation materials. (TIFF)

S4 Fig Study 2: Reminder cards. (TIFF)

S1 Table Robustness of results when controlling for canvasser. Given that randomization occurred at the shift stage, one concern would be that particular canvassers are “more effective” than others and that they might be assigned to a disproportionate number of pledge or control shifts. If this were to occur, then the findings we report in the paper may actually result from canvasser effectiveness rather than the treatment itself. Fortunately, DOF was able to supply us with the canvasser who contacted each subject in our experiment. In Colorado, there were 11 unique canvassers; in Pennsylvania, there were six. To account for the potential confounding effects of canvassers, we estimated the treatment effects using OLS models with canvasser fixed effects. The results from this alternative specification are presented here. The results in S1 Table indicate that our findings are robust to accounting for which canvasser recruited each subject. Whereas our main effect for Pennsylvania was 4.5 points among all eligible individuals and 8.8 points among those who had never voted, the estimates in S1 Table are 2.4 and 5.1 points, respectively. In Colorado, the treatment effects we present in the paper are 3.5 points for all eligible subjects and 5 points for those who had never voted, whereas those effects are 3.4 and 5.3 points in this analysis. Thus, even once accounting for potential canvasser effects, we still find that turnout is higher among those who received the pledge treatment.
(TIFF)

S2 Table Robustness of results when removing older individuals from analysis. Recruitment for this experiment happened on college campuses with the aim of mostly mobilizing young adults. However, recruiters contacted any individual that passed by, not just those who were younger in age. Accordingly, about 12% of the subjects who were contacted in Pennsylvania and for whom we have age information from the voter file were 30 years of age or older. In Colorado, about 8% of those contacted and matched to the voter file were 30 or older. S2 Table shows the treatment effects when we remove from the analysis any individuals who are 30 years of age or older. The effects here are for all individuals who were eligible to be treated. In Pennsylvania, limiting the analysis in this way results in a treatment effect that is somewhat larger than the 4.5 point effect reported in Table 2 and in Colorado we find a treatment effect that is identical to the 4.2 point effect that we report in Table 3.
(TIFF)
