# Supplementary figures and images for: Walking the walk? Experiments on the effect of pledging to vote on youth turnout
Source: PLoS One. 2018 May 29;13(5):e0197066. doi: 10.1371/journal.pone.0197066 (PMC5973556; doi:10.1371/journal.pone.0197066)

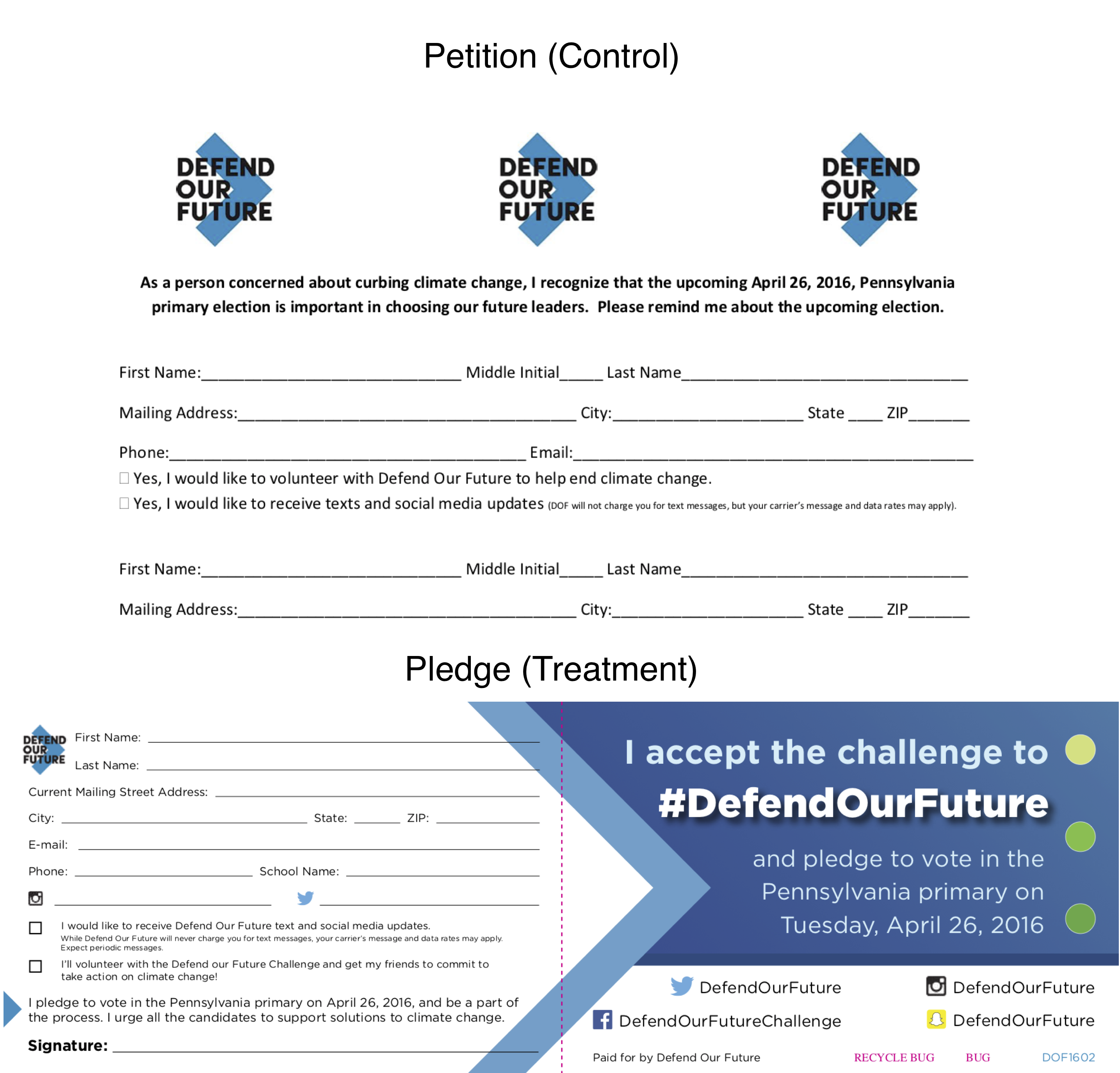

Supplement: S1 Fig — (TIFF) [file pone.0197066.s001.tiff]

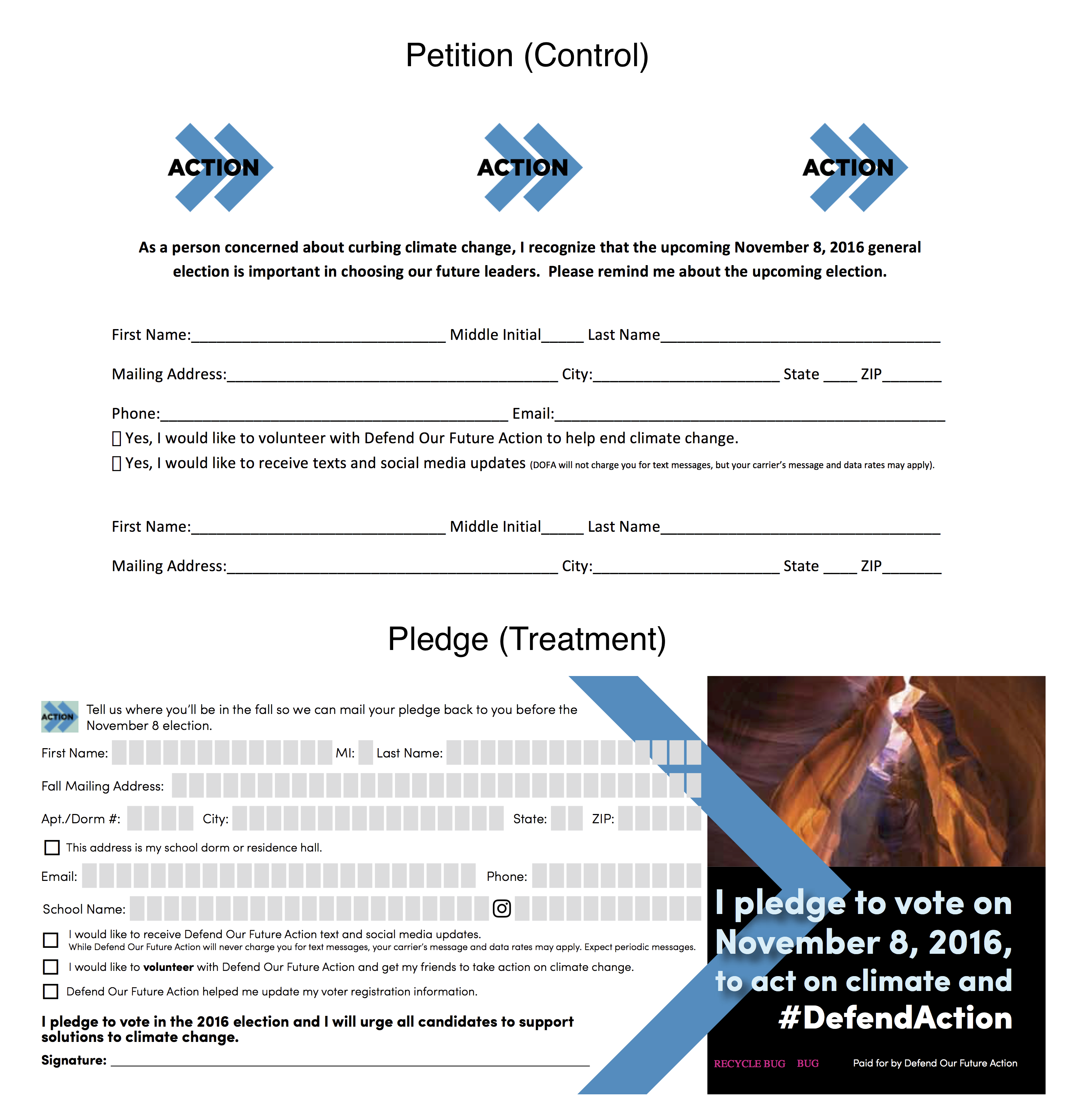

Supplement: S3 Fig — (TIFF) [file pone.0197066.s003.tiff]

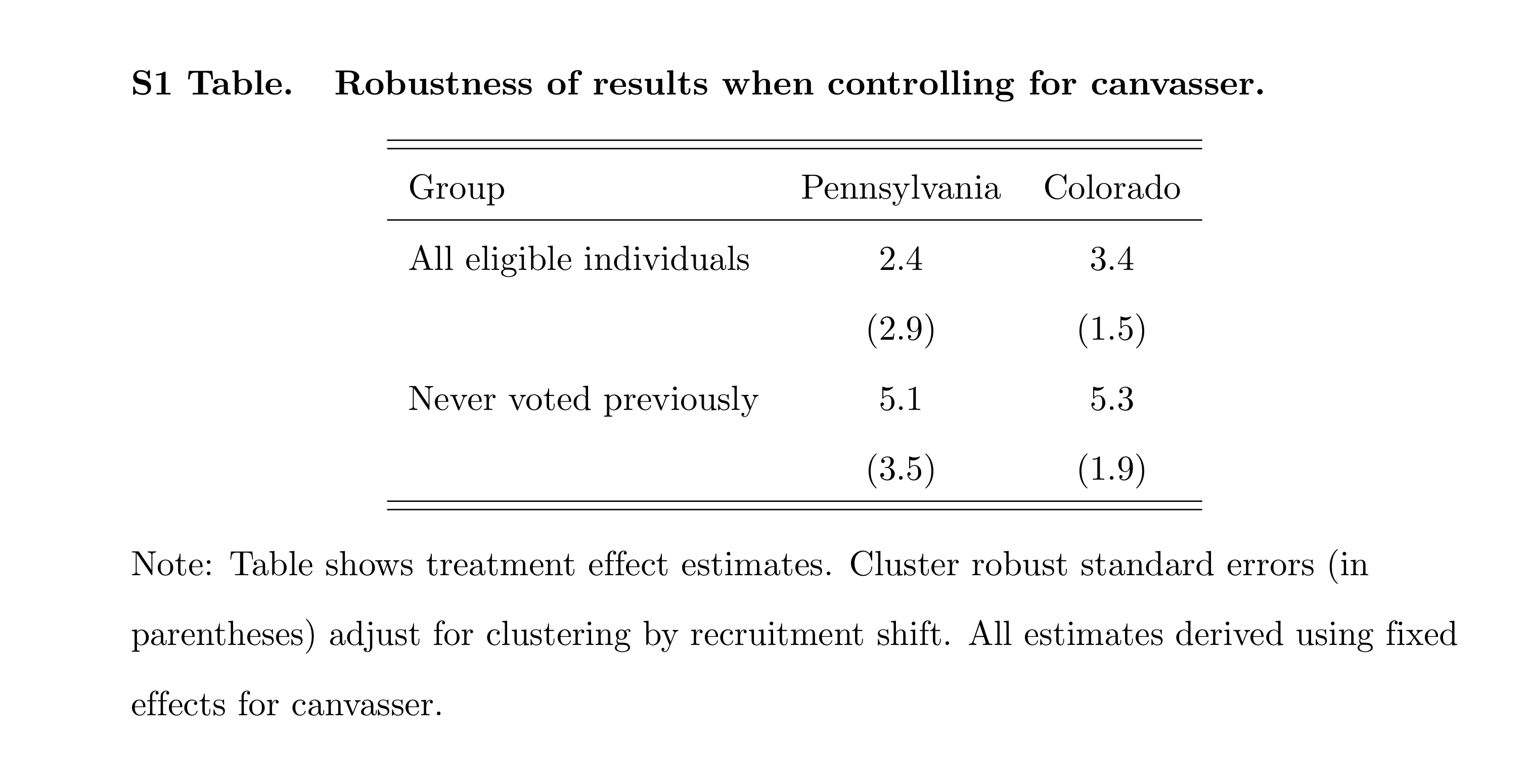

Supplement: S1 Table — Given that randomization occurred at the shift stage, one concern would be that particular canvassers are “more effective” than others and that they might be assigned to a disproportionate number of pledge or control shifts. If this were to occur, then the findings we report in the paper may actually result from canvasser effectiveness rather than the treatment itself. Fortunately, DOF was able to supply us with the canvasser who contacted each subject in our experiment. In Colorado, there were 11 unique canvassers; in Pennsylvania, there were six. To account for the potential confounding effects of canvassers, we estimated the treatment effects using OLS models with canvasser fixed effects. The results from this alternative specification are presented here. The results in S1 Table indicate that our findings are robust to accounting for which canvasser recruited each subject. Whereas our main effect for Pennsylvania was 4.5 points among all eligible individuals and 8.8 points among those who had never voted, the estimates in S1 Table are 2.4 and 5.1 points, respectively. In Colorado, the treatment effects we present in the paper are 3.5 points for all eligible subjects and 5 points for those who had never voted, whereas those effects are 3.4 and 5.3 points in this analysis. Thus, even once accounting for potential canvasser effects, we still find that turnout is higher among those who received the pledge treatment. (TIFF) [file pone.0197066.s005.tiff]

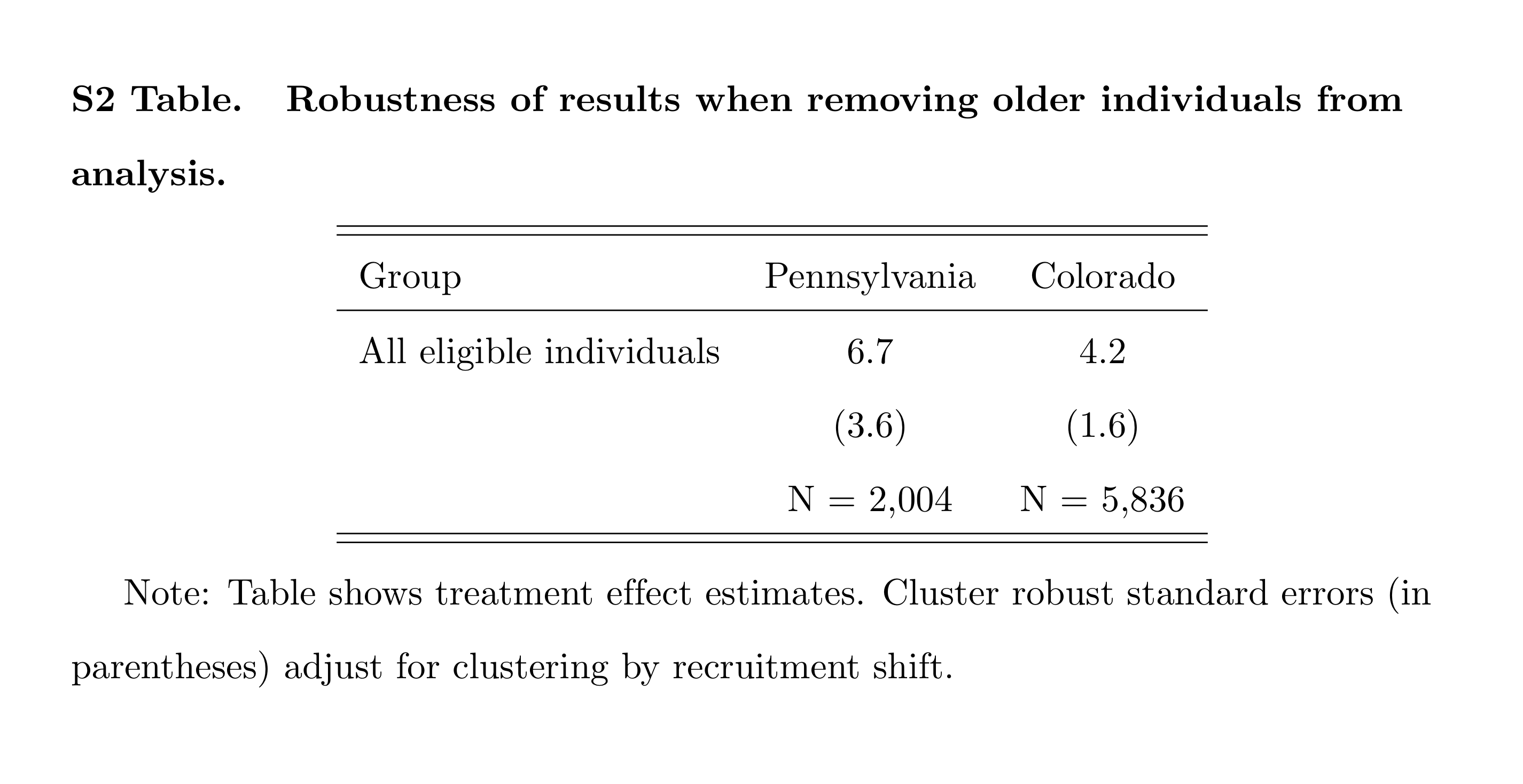

Supplement: S2 Table — Recruitment for this experiment happened on college campuses with the aim of mostly mobilizing young adults. However, recruiters contacted any individual that passed by, not just those who were younger in age. Accordingly, about 12% of the subjects who were contacted in Pennsylvania and for whom we have age information from the voter file were 30 years of age or older. In Colorado, about 8% of those contacted and matched to the voter file were 30 or older. S2 Table shows the treatment effects when we remove from the analysis any individuals who are 30 years of age or older. The effects here are for all individuals who were eligible to be treated. In Pennsylvania, limiting the analysis in this way results in a treatment effect that is somewhat larger than the 4.5 point effect reported in Table 2 and in Colorado we find a treatment effect that is identical to the 4.2 point effect that we report in Table 3. (TIFF) [file pone.0197066.s006.tiff]
